# 2019 Community-acquired Pneumonia Treatment Guidelines: There Is a Need for a Change toward More Parsimonious Antibiotic Use

**DOI:** 10.1164/rccm.201911-2226LE

**Published:** 2020-05-15

**Authors:** Benedikt Huttner, Bernadette Cappello, Graham Cooke, Sumanth Gandra, Stephan Harbarth, Monica Imi, Mark Loeb, Marc Mendelson, Lorenzo Moja, Céline Pulcini, Mike Sharland, Evelina Tacconnelli, Mei Zeng

**Affiliations:** ^1^ World Health Organization Geneva, Switzerland; ^2^ Geneva University Hospitals Geneva, Switzerland; ^3^ University of Geneva Geneva, Switzerland; ^4^ Imperial College London London, United Kingdom; ^5^ Washington University School of Medicine St Louis, Missouri; ^6^ Management Sciences for Health Kampala, Uganda; ^7^ McMaster University Hamilton, Ontario, Canada; ^8^ University of Cape Town Cape Town, South Africa; ^9^ Université de Lorraine Nancy, France; ^10^ St George’s University of London London, United Kingdom; ^11^ University of Verona Verona, Italy and; ^12^ Children’s Hospital of Fudan University Shanghai, China

*To the Editor*:

The American Thoracic Society (ATS) and the Infectious Diseases Society of America (IDSA) recently published updated guidelines for the diagnosis and treatment of adults with community-acquired pneumonia (CAP) (1). In the 12 years since the previous edition of the guidelines was published, the importance of incorporating antimicrobial stewardship principles into treatment guidelines has been increasingly recognized (2). In 2017, the U.S. Healthcare Infection Control Practices Advisory Committee provided guidance regarding this issue for U.S. treatment guidelines. One of the recommendations states that “when multiple therapeutic options are available, a hierarchy of antibiotic treatment recommendations should be provided with ‘first choice’ options being those with adequate therapeutic efficacy, the lowest risk of facilitating antimicrobial resistance, and the lowest risk of promoting *C. difficile* and other adverse events, with consideration of healthcare value” (3). The World Health Organization (WHO) recently developed the AWaRe (Access, Watch, and Reserve) framework for classifying antibiotics based on antibiotic stewardship principles, and recommends its use in treatment guidelines (4, 5).

We are therefore concerned that the 2019 version of the ATS/IDSA CAP guidelines seems to give disappointingly little weight to such antibiotic stewardship principles while continuing to recommend WHO Watch and Reserve antibiotics as first-line options for CAP in most of the target populations. We suggest that Access antibiotics would be sufficient for many patients and would be preferable from an antibiotic stewardship perspective. Amoxicillin, which is the first-choice treatment for CAP based on the 2019 WHO Model List of Essential Medicines, and is also listed as a first-choice option in many guidelines outside the United States, is only recommended in the ATS/IDSA guidelines, together with doxycycline and macrolides as equivalent options, for patients without comorbidities (very broadly stated as “chronic heart, lung, liver, or renal disease; diabetes mellitus; alcoholism; malignancy; or asplenia”) (6, 7).

In the United States, 6 in 10 adults have one chronic disease, and 4 in 10 adults have two or more chronic diseases, so the recommendation for amoxicillin will be applicable to a minority of adults with CAP (8). Therefore, U.S. physicians will most likely continue to treat many patients with the Watch group respiratory fluoroquinolones, a class of antibiotics with a well-documented propensity to favor the emergence and spread of antibiotic resistance and *Clostridioides difficile* infections, as well as an increased risk of adverse events (U.S. Food and Drug Administration alert) (9, 10).

This will hamper the CDC’s efforts to reduce overall antibiotic consumption and fluoroquinolone use in the United States (11). The Healthcare Infection Control Practices Advisory Committee also suggested that guidelines should include recommendations to educate patients about antibiotic therapy when appropriate. Accordingly, the developers of the CAP guidelines could have considered providing guidance to physicians regarding the use of Access group antibiotics such as amoxicillin and doxycycline in select patients with stable comorbid conditions, along with close monitoring and adequate patient education.

Even more surprising is the listing of the Reserve group fifth-generation cephalosporin ceftaroline as a first-choice empiric treatment option for CAP (in combination with a macrolide) in hospitalized adults without risk factors for methicillin-resistant *Staphylococcus aureus* and *Pseudomonas aeruginosa*. Efficacy data from phase III trials suggesting that ceftaroline is superior to ceftriaxone with regard to clinical cure require further scrutiny, and its current listing as a first-choice option violates basic antibiotic stewardship considerations (12, 13).

Given that respiratory tract infections are one of the most frequent reasons for antibiotic prescriptions worldwide, and that in many countries the U.S. treatment recommendations are still considered an important reference, it seems to us, as members of the WHO EML Antibiotics Working Group, that this represents a lost opportunity for antibiotic stewardship. We believe there is a clear need to better align all treatment guidelines to the same guiding principles, and to establish a global set of evidence-based recommendations with a focus on enhancing the use of Access group antibiotics.

## Supplementary Material

Supplements

Author disclosures
