# Supplementary material for: 2019 Community-acquired Pneumonia Treatment Guidelines: There Is a Need for a Change toward More Parsimonious Antibiotic Use
Source: Am J Respir Crit Care Med. 2020 May 15;201(10):1315–6. doi: 10.1164/rccm.201911-2226LE (PMC7233347; doi:10.1164/rccm.201911-2226LE)
Supplement: Supplements [file rccm.201911-2226LE.html]

2019 Community-acquired Pneumonia Treatment Guidelines: There Is a Need for a Change toward More Parsimonious Antibiotic Use | American Journal of Respiratory and Critical Care Medicine

- disclosures.pdf (369 KB)
